# User Perceptions and Use of an Enhanced Electronic Health Record in Rwanda With and Without Clinical Alerts: Cross-sectional Survey

**DOI:** 10.2196/32305

**Published:** 2022-05-03

**Authors:** Hamish S F Fraser, Michael Mugisha, Eric Remera, Joseph Lune Ngenzi, Janise Richards, Xenophon Santas, Wayne Naidoo, Christopher Seebregts, Jeanine Condo, Aline Umubyeyi

**Affiliations:** 1 Brown Center for Biomedical Informatics Brown University Providence, RI United States; 2 School of Public Health University of Rwanda Kigali Rwanda; 3 Ministry of Health Kigali Rwanda; 4 Centers for Disease Control Atlanta, GA United States; 5 Jembi Health Systems Cape Town South Africa

**Keywords:** electronic health record, eHealth, HIV/AIDS, survey, Rwanda, implementation science

## Abstract

**Background:**

Electronic health records (EHRs) have been implemented in many low-resource settings but lack strong evidence for usability, use, user confidence, scalability, and sustainability.

**Objective:**

This study aimed to evaluate staff use and perceptions of an EHR widely used for HIV care in >300 health facilities in Rwanda, providing evidence on factors influencing current performance, scalability, and sustainability.

**Methods:**

A randomized, cross-sectional, structured interview survey of health center staff was designed to assess functionality, use, and attitudes toward the EHR and clinical alerts. This study used the associated randomized clinical trial study sample (56/112, 50% sites received an enhanced EHR), pulling 27 (50%) sites from each group. Free-text comments were analyzed thematically using inductive coding.

**Results:**

Of the 100 participants, 90 (90% response rate) were interviewed at 54 health centers: 44 (49%) participants were clinical and 46 (51%) were technical. The EHR top uses were to access client data easily or quickly (62/90, 69%), update patient records (56/89, 63%), create new patient records (49/88, 56%), generate various reports (38/85, 45%), and review previous records (43/89, 48%). In addition, >90% (81/90) of respondents agreed that the EHR made it easier to make informed decisions, was worth using, and has improved patient information quality. Regarding availability, (66/88) 75% said they could *always or almost always* count on the EHR being available, whereas (6/88) 7% said *never/almost never*. In intervention sites, staff were significantly more likely to update existing records (*P*=.04), generate summaries before (*P*<.001) or during visits (*P*=.01), and agree that “the EHR provides useful alerts, and reminders” (*P*<.01).

**Conclusions:**

Most users perceived the EHR as well accepted, appropriate, and effective for use in low-resource settings despite infrastructure limitation in 25% (22/88) of the sites. The implementation of EHR enhancements can improve the perceived usefulness and use of key functions. Successful scale-up and use of EHRs in small health facilities could improve clinical documentation, care, reporting, and disease surveillance in low- and middle-income countries.

## Introduction

### Background

Effective and high-quality health care requires high-quality, timely health information—“Information is care” [1]. Scaling up effective care for millions of patients with HIV in resource-limited settings such as sub-Saharan Africa required the development of new paradigms for the collection, storage, viewing, and analysis of clinical data and health information [2]. Most health centers treating HIV started with only structured paper records. As the volume of patient data grew and in-country digital capacity improved, electronic tools were introduced. Many early electronic health records (EHRs) in resource-limited settings have been developed for HIV care, including those in Malawi [3], Kenya [4], and Haiti [5]. These projects demonstrated the feasibility of deploying health information systems, improvements in reporting to ministries of health (MoHs) and donors, and the ability to monitor the continuum of care. Furthermore, this initial evidence also suggested that the use of EHR systems for HIV, tuberculosis (TB) and multidrug-resistant TB treatment could improve the quality of care [2]. A critical challenge to improving the quality of care in low-income settings is the ability to achieve long-term, consistent EHR use at a large scale. To better understand the perceptions and clinical uses of EHR systems that support improved use and care in Rwanda, we conducted a quantitative user survey supplemented by free-text questions. For the purposes of this study, the terms *electronic health record* and *electronic medical record* are used interchangeably.

### HIV Care in Rwanda

Rwanda is an East Central African country bordering Tanzania, Uganda, Burundi, and the Democratic Republic of the Congo. Rwanda had a per capita income of US $773 in 2018, up from US $241 in 2004 [6], and has made great progress in rebuilding its health care systems after the genocide against Tutsi in 1994. A major health challenge Rwanda has faced, along with neighboring countries in Africa, is the HIV epidemic. A 2018 to 2019 survey indicated that HIV prevalence among adults aged 15 to 49 years was 2.6% [7]. Great strides have been made in the treatment of patients who are HIV positive, including improvements in the prevention of mother-to-child transmission uptake, and reduction in the rate of loss to follow-up for patients receiving antiretroviral therapy (ART) in Rwanda. This is demonstrated by the near achievement in 2019 of the 2020 Joint United Nations Programme on HIV/AIDS 90-90-90 goal, with 84% of adults who were HIV positive knowing their status, 98% of those knowing their status on ART, and 90% of those on ART having a suppressed viral load [7]. From the beginning of the HIV treatment scale-up, the Government of Rwanda has emphasized care and prevention in rural areas as well as in urban settings; recruitment, training, and supervision of community health workers; and the use of health information systems. These information systems included national-level surveillance systems for HIV care [8,9], mobile health systems to support antenatal and primary care, and patient information or EHR systems mainly for supporting HIV care in health centers and hospitals. The 3 main EHR systems used have been OpenMRS (OpenMRS Inc) in health centers and 36 district hospitals offering HIV services, IQcare (International Quality Care, Palladium Inc) [10] in some health centers (now replaced by OpenMRS), and OpenClinic (OpenClinic GA) [11] in some hospitals. Since 2009, the MoH has moved to using OpenMRS for all HIV health centers and most hospitals in the country.

### OpenMRS

OpenMRS is an open-source software platform for building EHRs, with a focus on health care needs in low- and middle-income countries (LMICs). Founded in 2004, the OpenMRS community set goals to create a public software platform to assist health care organizations worldwide in developing EHR systems that were adaptable to local needs, owned by local organizations, and programmed by local developers as much as possible [12] (Textbox 1).

**The OpenMRS electronic health record system.**

OpenMRS has an unusual modular architecture allowing modules from the core development team to be mixed with modules from other developers to create flexible and updatable systems, with typical implementations using 35 to 45 modules. This ensures the core OpenMRS code is common to nearly all OpenMRS installations. Data are stored using a concept dictionary allowing flexibility in data capture and translation to other languages [12]. This approach also supports a range of standards for data storage and exchange with mappings available for a range of coding standards such as the International Classification of Diseases, 10th Revision, and Logical Observation Identifiers Names and Codes in the master Columbia International eHealth Laboratory concept dictionary.

Adapting OpenMRS to new uses typically requires technical expertise including Java programming if new modules are required. There were limitations to the older user interface used in this project (which has now been superseded), requiring care in developing clinical workflows. OpenMRS has been adapted to support a wide range of care including HIV, multidrug-resistant tuberculosis, primary care, emergency care, heart disease, oncology, and surgery. A Server Monitoring Tool module was developed to track system uptime and downtime, daily data entry rates, and completeness of key variables. The Server Monitoring Tool was used as part of the larger evaluation study in Rwanda.

OpenMRS was developed by a collaboration among the Academic Model Providing Access to Healthcare project in Kenya with the Regenstrief Institute in Indiana, United States; the Partners In Health Informatics team in Rwanda and Boston, Massachusetts, United States (HSF); and the informatics lead of the South African Medical Research Council (now CEO of Jembi Health Systems, Cape Town, South Africa—CS). Ongoing maintenance of the core OpenMRS platform is accomplished through the OpenMRS community—a worldwide network of volunteers with technology, health care, and international development expertise.

Initially, OpenMRS was used for HIV and TB treatment in outpatient settings, supporting projects funded by the US President’s Emergency Plan for AIDS Relief and the Global Fund for AIDS, Tuberculosis, and Malaria. Currently, it covers a wide range of clinical areas. Partners In Health implemented and currently supports OpenMRS in 46 health centers and 3 hospitals in Rwanda covering HIV care, pediatrics, primary care, cardiology, and oncology.

Between 2009 and 2013, the Rwanda MoH deployed OpenMRS to >300 health centers providing HIV care throughout the country [13]. Before and during deployment, OpenMRS had to be customized to support the Rwanda MoH requirements. A dedicated 9-month course led by Partners In Health/Inshuti Mu Buzima trained programmers in enterprise Java and health information system design [14]. Several graduates were hired by the MoH and created custom OpenMRS modules for HIV and primary care using OpenMRS version 1.6 core code. This is the version of OpenMRS used in the control sites for this study. Unstable internet connectivity in rural Rwanda (similar to many low-income countries) required each site to run its own instance on a local server, requiring stable power and local technical support.

### Impact of EHR Systems in Resource-Limited Countries

Over the last two decades, EHR systems have been implemented in a wide range of countries, including those with the lowest income levels. The scale-up of HIV care and transition from an emergency outbreak response to a lifelong chronic care model was a major driver for the expansion of EHR system use and the development of common, shared information system tools. Countries, including Rwanda, Kenya, Uganda, Mozambique, and Nigeria, have scaled up the use of OpenMRS EHR systems for HIV care to hundreds of their clinical sites. Other EHRs, including IQcare, have been widely used in countries such as Kenya [15].

The OpenMRS community has prioritized support for effective and safe clinical care as well as reporting and research. Smaller-scale studies have evaluated the impact of EHR system improvements on the aspects of clinical care, the systems *efficacy*. Were et al [16] studied the addition of alerts to printed patient summaries generated by OpenMRS on a range of clinical actions for the care of children who were HIV positive in Eldoret, Kenya. In a randomized controlled trial (RCT), they showed that health care workers receiving the summaries with alerts were 4 times more likely to carry out actions such as ordering CD4 counts (a T lymphocytes test) and polymerase chain reaction tests for HIV antigen. In a larger study, Oluoch et al [17] studied the impact of improved decision support tools implemented in an EHR in Kenya on the quality of HIV care. In a cluster RCT of 13 health centers and 41,062 patients, they showed that sites with the decision support tools were quicker and more effective in responding to HIV treatment failure [17]. Critical questions remain regarding the key factors that determine individual EHR use, facilitate scaling up to tens or hundreds of smaller health facilities, support long-term use, and influence the clinical impact of these systems in routine care—the *effectiveness* of EHRs in LMICs [18].

## Methods

### Overview

The aims of this study are to evaluate the following questions in a large number of health centers in Rwanda: (1) staff and stakeholder expectations and perceptions of health information system performance; (2) staff and stakeholder expectations and perceptions around effort expended to use health information systems; (3) infrastructural, organizational, and individual conditions that are barriers and facilitators to using such tools (including training and technical support); (4) staff perceptions of technology fatigue; and (5) any differences in the experiences of staff in intervention and control sites and between clinical and technical users.

### The EHR Implementation Science Study

The focus of this manuscript is the electronic medical record (EMR) user survey component of a process evaluation, which is part of a larger, 3-part implementation science study on the use of an *enhanced* EHR to support HIV care in 56 randomly allocated health centers that commenced in July 2018. It included the evaluation of (1) EHR use, performance, and data quality; (2) the clinical impact in an RCT; and (3) the cost of development and implementation of the enhanced EHR functionality.

For enrollment in the overall study, first, the enhanced EHR package (Textbox 2) was piloted in 2 health centers in Kigali (Kicukiro Health Centre and Kagugu Health Centre), and improvements were made in response to the user experience and comments. Next, the following selection criteria were applied: (1) the presence of ≥3 computers, 1 printer, and a local area network; (2) active HIV case numbers between 50 and 700; and (3) successful installation of the Server Monitoring Tool (Textbox 1) and evidence of regular data entry by staff. Using these criteria, a total of 112 sites were selected to participate in the clustered RCT. These sites were a mix of urban and rural health centers and some district hospitals. Of the 112 sites, 56 (50%) were randomized into the intervention sites, which had the enhanced EHR installed on the servers between June 25 and July 5, 2018. All 56 sites had the alerts for delayed patient enrollment, 28 sites also had alerts for delayed viral load testing, and 14 had the alerts for evidence of treatment failure. For the analysis of the survey, sites with at least the top-level alerts (delayed HIV care registration) were classed as *intervention*. Health facility staff, including clinicians, data managers, local information technology (IT) staff, local clinic managers, and district IT specialists, in all 112 study sites were trained on general EHR use and data management. Additional training was provided for staff in the intervention sites on the enhanced EHR and equivalent training on the control EHR.

**The enhanced electronic health record (EHR) package.**

**The enhanced EHR package enhancements**

- Upgraded OpenMRS software version (to v.1.11) and additions to the concept dictionary
- Improved workflow for registering and managing patients with HIV
- Improved ordering of laboratory analyses (HIV tests, CD4 counts, and viral loads)
- Upgraded clinician summaries of patients showing key clinical data and alerts and reminders designed to improve care
- Custom automatic reports to identify patients not receiving optimal care, implementing the same alerts and reminders
- Alerts and reports designed to identify patients with care delivery issues. These were chosen to reflect the needs identified by the Rwandan Ministry of Health and based on the 2016 World Health Organization guidelines for HIV care (WHO Consolidated Guidelines HIV 2016 [19]) and included the following:
  - Newly diagnosed patients with HIV who have not been enrolled in antiretroviral therapy within 2 weeks of diagnosis
  - Patients with 8 months of antiretroviral therapy who do not have a viral load test result in the EHR (6 months of care + 2 months for result to return and be entered in the EHR)
  - Patients who have an abnormal (elevated) viral load result and require assessment and management for treatment failure

#### Study Environment

The user survey was conducted at primary health care facilities, referred to here as health centers, offering HIV treatment services, located throughout Rwanda, approximately 5 months after the installation of the enhanced EHR.

#### Study Design

This study used a cross-sectional, key informant structured interview design within control and intervention sites. The data were collected through structured interviews to ensure high response rates and avoid technical limitations that may have impacted a web-based survey and biased results toward better-supported sites and users. The goal is to gain insights into the adoption, functionality, use, and perceptions of EHRs by clinical staff (nurses, physicians, and social workers) and technical staff (IT staff, data entry staff, and data managers) in health centers. Care of patients in smaller health facilities in East Africa, including those with HIV, is mostly carried out by nurses or junior clinician grades and rarely by physicians. The study questions were as follows: (1) whether the actions and perceptions of staff using the enhanced EHR intervention would be different from those using the control EHR and (2) whether clinicians have different experiences with the EHR than technical staff.

#### Sampling and Sample Size

**Figure 1:**
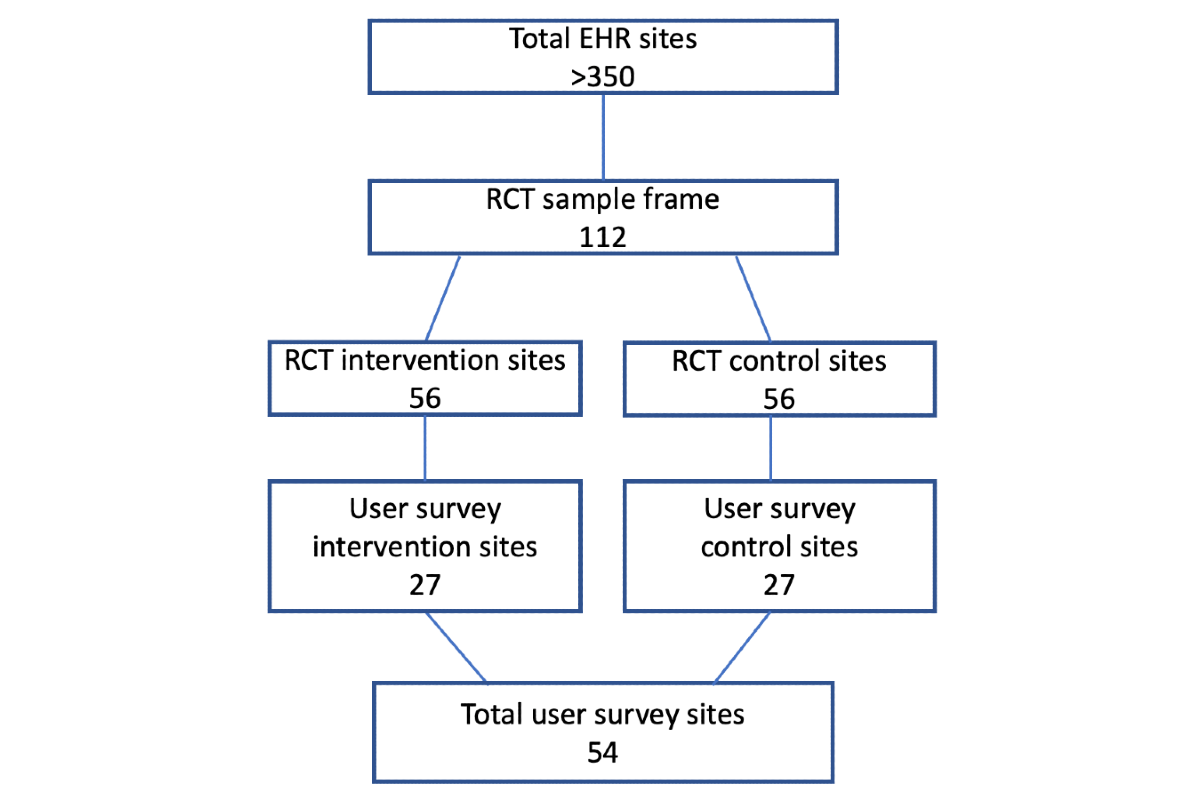
Sample design (total electronic medical record sites include those managed by Partners In Health/Inshuti Mu Buzima; there were >300 active ministry of health–run sites). EHR: electronic health record; RCT: randomized controlled trial.

This study drew from the sample frame of the clustered RCT implementation study. The RCT enrolled 112 health centers from >300 that use the OpenMRS EHR for HIV care. Of the 112 sites, 54 (48.2%) were randomly selected, including 27 (50%) from the enhanced EHR sites (intervention) and 27 (50%) control sites (Figure 1). Randomization was performed with R.

A total of 100 participants were approached for the structured interview, with the goal of 1 clinician (nurse or physician) and 1 data manager at each health center. If not available, other EHR users were recruited if possible.

#### Data Collection Tool

The structured interview and observation (survey) tool included sections on demographics, experience with IT, EHR training received, frequency of EHR use for different tasks, overall ease of use, usefulness for specific tasks, technical and user support, and system stability and infrastructure issues. The survey tool included 5-point Likert scale quantitative close-ended questions and qualitative open-ended questions. It was adapted from a form originally used by Médecins Sans Frontières, piloted in 20 clinics in Rwanda in 2012 [13], and translated from English into Kinyarwanda.

#### Data Collection

The survey was conducted by 10 trained data collectors from the Rwanda School of Public Health. Survey responses were documented and recorded in a preprogrammed Android tablet using ODK [20]. Free-text comments were documented in Kinyarwanda, translated into English, and reviewed by bilingual research team members before analysis. Written informed consent was obtained, and participants’ confidentiality was assured using a private interview room at each health center surveyed.

#### Data Analysis

Descriptive statistics were carried out using Excel (Microsoft Corp). JMP Statistical Software (SAS Institute) and Excel were used for the chi-square tests for the 5-point Likert scale responses. For the comparison of clinicians and technical users, all Likert scale questions were tested for significance. For the comparison of the intervention and control sites, the 2 groups of questions (12 and 18) most directly related to the technical improvements in the enhanced EHR were tested. *P* values were adjusted for multiple comparisons using the Benjamini and Hochberg method and R p.adjust [21]. Analyses were designed and carried out with assistance from a statistician and a data scientist at Brown University (see *Acknowledgments*).

Free-text comments, which were all short statements, were analyzed thematically using inductive coding by one author (HSF) and recoding by a second author (MM), with discrepancies resolved by discussion. Common concepts were described and rated based on the number of user responses matching each code.

### Ethical Considerations

This study was approved by the following investigational review boards: Rwanda National Ethics Committee, Kigali (#913/RNAC/2016) and the University of Leeds School of Medicine Research Ethics Committee, Leeds, United Kingdom (MREC16-176). This study was reviewed in accordance with the US Centers for Disease Control and Prevention human research protection procedures (approval #CGH HSR 2014-270a) and determined to be research. However, investigators of the Centers for Disease Control and Prevention did not interact with human participants or have access to identifiable data during this research.

## Results

### Participant Characteristics

A total of 100 participants were approached for interview and consented to participate. Of these 100 participants, 90 (90% response rate) were available at the time of the visit by study staff, with 44 (49%) from the intervention sites. The participants had a mean age of 35.9 (SD 6.2; range 23-58) years, a mean of 7.5 (SD 2.0; range 0-38) years working at the health centers, and 47% (42/90) were female. Their educational attainment was reported as *some secondary schooling* (8/90, 9%), *completed secondary schooling* (18/90 20%), and *postsecondary schooling* (64/90, 71%). Their occupations were nurse (41/90, 46%), physician (1/90, 1%), social worker (2/90, 2%), data manager (42/90, 47%), IT officer (1/90, 1%), and data entry staff (3/90, 3%). Respondents had a mean of 3.3 (SD 2.0) years of experience with the EHR and a mean of 1.9 (SD 1.4) trainings and 9.7 (SD 7.9) training days.

### General Use of Technology

A large majority of respondents used mobile phones, with 82% (74/90) using them “about half the time,” “most of the time,” or “all of the time” for texting, and 82% (74/90) similarly for mobile data. Computer use outside of work was reported by 31% (28/90) and internet use by 49% (44/90). Clinicians (physicians, nurses, and social workers) reported significantly less use than technical staff (IT officers, data managers, and data entry staff; *P*=.009 and *P*=.04, respectively).

### Training on EHR

Respondents agreed or strongly agreed that their training on the EHR was effective (82/84, 98%), and they were confident in using the EHR (81/87, 93%). However, 77% (66/86) of respondents disagreed or strongly disagreed with the statement “I am generally not concerned making errors in EHR.” There were no statistically significant differences in responses on training between clinicians and technical staff. However, in free-text comments, 81% (73/90) of respondents requested more training. These requests included refreshers, training on new modules or updates, and more practical hands-on training. There were also requests for training in reports and data analysis. Mentorship, supportive supervision, or more technical backup were requested by many respondents.

### Use of EHR Functions

Tables 1-7 show and summarize the results for the following question: “Please indicate how often you use the EMR to assist you with the following tasks.” Combining the categories most of the occasions and always/almost always, the percentages for common tasks were 56% (49/88) for creating new patient records, 63% (56/89) for updating existing patient records, 40% (36/89) for generating patient summaries before visits, 48% (43/89) for reviewing previous patient encounters, 30% (21/69) for ordering laboratory analyses, 43% (36/83) for viewing laboratory results, 33% (25/75) for following test results over time, 45% (38/85) for generating automatic reports, 45% (38/85) for generating ad hoc reports (eg, quarterly or TracNET reports), and 49% (41/84) for referring patients to another health facility. The results were 22% (18/82) for generating consult sheets and 16% (14/85) for generating clinician summaries.

**Table 1:** Frequency of survey responses for Likert scale data: question 6 (n=90).

Question 6. How often do you do the following activities?	1—never/almost never, n (%)	2—seldom, n (%)	3—about half the occasions, n (%)	4—most of the occasions, n (%)	5—always/almost always, n (%)	Top 2 groups, n (%)
Use a mobile phone to send text messages	5 (6)	3 (3)	8 (9)	40 (44)	34 (38)	74 (82)
Use a mobile phone to access email, internet, WhatsApp, or Facebook	5 (6)	2 (2)	9 (10)	44 (49)	30 (33)	74 (82)
Use a computer outside of work	28 (31)	5 (6)	29 (32)	16 (18)	12 (13)	28 (31)
Access the internet to check email, go to websites, or any other internet activities	13 (14)	13 (14)	20 (22)	27 (30)	17 (19)	44 (49)

**Table 2:** Frequency of survey responses for Likert scale data: question 10.

Question 10. Training	Strongly disagree, n (%)	Disagree, n (%)	Neutral, n (%)	Agree, n (%)	Strongly agree, n (%)		Top 2 groups, n (%)
The training I received relating to the EMR^a^ was effective (n=84)	1 (1)	1 (1)	0 (0)	21 (25)	61 (73)		82 (98)
In general I am not concerned about making errors in the EMR (n=86)	36 (42)	30 (35)	3 (3)	13 (15)	4 (5)	17 (20)	
I am confident using the EMR (n=87)	1 (1)	4 (5)	1 (1)	42 (48)	39 (45)		81 (93)

^a^EMR: electronic medical record.

**Table 3:** Frequency of survey responses for Likert scale data: questions 12 to 14.

Question		Never/almost never, n (%)	Seldom, n (%)	About half of the occasions, n (%)	Most of the occasions, n (%)	Always/almost always, n (%)	Top 2 groups, n (%)
**12. Please indicate how often you use the electronic medical record to assist you with the following tasks.**							
	Creating new patient records (n=88)	2 (2)	14 (16)	23 (26)	17 (19)	32 (36)	49 (56)
	Updating existing patient records (n=89)	3 (3)	10 (11)	20 (22)	26 (29)	30 (34)	56 (63)
	Generating patient summaries before visits (n=89)	11 (12)	14 (16)	28 (31)	21 (24)	15 (17)	36 (40)
	Reviewing previous patient encounters (n=89)	6 (7)	13 (15)	27 (30)	21 (24)	22 (25)	43 (48)
	Ordering laboratory analyses (n=69)	32 (46)	5 (7)	11 (16)	12 (17)	9 (13)	21 (30)
	Viewing laboratory results (n=83)	22 (27)	7 (8)	18 (22)	19 (23)	17 (20)	36 (43)
	Following test results over time (n=75)	32 (43)	4 (5)	14 (19)	15 (20)	10 (13)	25 (33)
	Ordering medicine (n=65)	50 (77)	3 (5)	5 (8)	2 (3)	5 (8)	7 (11)
	Generating pharmacy reports (n=79)	54 (68)	4 (5)	7 (9)	7 (9)	7 (9)	14 (18)
	Generating automatic reports (n=85)	31 (36)	8 (9)	8 (9)	14 (16)	24 (28)	38 (45)
	Generating ad hoc reports (n=85)	34 (40)	2 (2)	11 (13)	13 (15)	25 (29)	38 (45)
	Generating consult sheets (n=82)	47 (57)	5 (6)	12 (15)	12 (15)	6 (7)	18 (22)
	Generating clinician summaries (n=85)	48 (56)	8 (9)	15 (18)	8 (9)	6 (7)	14 (16)
	Referring patients to another health center (n=84)	20 (24)	6 (7)	17 (20)	14 (17)	27 (32)	41 (49)
13. All considered, how often do you use the electronic medical record as an information source in your clinical work? (n=89)		13 (15)	10 (11)	29 (33)	38 (40)	1 (1)	37 (42)
14. All considered, how often do you use paper-based medical records as an information source in your clinical work? (n=89)		4(4)	4 (4)	13 (15)	61 (69)	7 (8)	68 (76)

**Table 4:** Frequency of survey responses for Likert scale data: question 16.

Question 16. Please tell us the degree to which you agree or disagree with the following statements about the EMR^a^.	Strongly disagree, n (%)	Disagree, n (%)	Neutral, n (%)	Agree, n (%)	Strongly agree, n (%)	Top 2 groups, n (%)
I am able to find where to document care (n=84)	6 (7)	3 (4)	4 (5)	49 (58)	22 (26)	71 (85)
In general it is easy to correct errors in EMR (n=89)	4 (4)	19 (21)	2 (2)	44 (49)	20 (22)	64 (72)
In general the screen display is easy to read (n=89)	1 (1)	2 (2)	1 (1)	38 (43)	47 (53)	85 (96)
The content is laid out in an understandable way (n=89)	1 (1)	6 (7)	5 (6)	53 (60)	24 (27)	77 (87)
It is easy to retrieve patient records in the EMR (n=89)	1 (1)	4 (4)	1 (1)	44 (49)	39 (44)	83 (93)

^a^EMR: electronic medical record.

**Table 5:** Frequency of survey responses for Likert scale data: question 18.

Question 18. Please tell us the degree to which you agree or disagree with the following statements about the EMR^a^.	Never/almost never, n (%)	Seldom, n (%)	About half of the occasions, n (%)	Most of the occasions, n (%)	Always/almost always, n (%)	Top 2 groups, n (%)
The EMR provides useful alerts, reminders (n=82)	5 (6)	7 (9)	6 (7)	36 (44)	28 (34)	64 (78)
The EMR makes it easier to manage patients (n=90)	0 (0)	2 (2)	1 (1)	37 (41)	50 (56)	87 (97)
The EMR easier to make informed decisions (n=90)	1 (1)	3 (3)	1 (1)	40 (44)	45 (50)	85 (94)
The EMR makes it easier exchange patient information with other health care providers (n=90)	0 (0)	21 (23)	5 (6)	34 (38)	30 (33)	64 (71)
The EMR is worth the time and energy to use (n=90)	0 (0)	1 (1)	0 (0)	44 (49)	45 (50)	89 (99)
The quality of information has improved due to the EMR (n=90)	0 (0)	3 (3)	4 (4)	50 (56)	33 (37)	83 (92)

^a^EMR: electronic medical record.

**Table 6:** Frequency of survey responses for Likert scale data: question 20.

Question 20. Please tell us the degree to which you agree or disagree with the following statements about the EMR^a^.	Strongly disagree, n (%)	Disagree, n (%)	Neutral, n (%)	Agree, n (%)	Strongly agree, n (%)	Top 2 groups, n (%)
It is easy to report problems with the EMR (n=89)	7 (8)	17 (19)	2 (2)	45 (51)	18 (20)	63 (71)
I get feedback when I report errors or problems with the EMR (n=89)	7 (8)	23 (26)	6 (7)	45 (51)	8 (9)	53 (60)
Effective help is available when I experience problems with the EMR (n=89)	9 (10)	28 (31)	3 (3)	40 (45)	9 (10)	49 (55)
I use the EMR because of the proportion of coworkers who use it (n=86)	11 (13)	37 (43)	3 (3)	27 (31)	8 (9)	35 (41)
My supervisor is very supportive of use of the EMR for my job (n=89)	7 (8)	9 (10)	6 (7)	39 (44)	28 (31)	67 (75)
In general, the Ministry of Health has supported the use of the EMR (n=90)	1 (1)	2 (2)	3 (3)	49 (54)	35 (39)	84 (93)

^a^EMR: electronic medical record.

**Table 7:** Frequency of survey responses for Likert scale data: question 22.

Question 22. Indicate how often you experience the following:	Never/almost never, n (%)	Seldom, n (%)	About half of the occasions, n (%)	Most of the occasions, n (%)	Always/almost always, n (%)	Top 2 groups, n (%)
How often can you count on EMR^a^ to be up and available? (n=88)	2 (2)	4 (5)	16 (18)	29 (33)	37 (42)	66 (75)
How often is grid electricity present? (n=90)	3 (3)	3 (3)	10 (11)	38 (42)	36 (40)	74 (82)
How often is the backup generator available? (n=88)	52 (59)	3 (3)	3 (3)	4 (5)	26 (30)	30 (34)
How often is there internet? (n=89)	12 (13)	2 (2)	19 (21)	20 (22)	36 (40)	56 (63)
How often is there cellular network coverage? (n=85)	27 (32)	6 (7)	11 (13)	13 (15)	28 (33)	41 (48)
How often is a computer available when you need to use the EHR^b^? (n=89)	4 (4)	2 (2)	6 (7)	12 (13)	65 (73)	77 (87)
How often is the EHR very slow? (reverse scale; n=88)	31 (35)	15 (17)	29 (33)	9 (10)	4 (5)	13 (15)

^a^EMR: electronic medical record.

^b^EHR: electronic health record.

Staff in intervention sites were significantly more likely to use the EHR for “Updating existing patient records” (*P*=.04), “Generating patient summaries before visits” (*P*<.001), “Viewing laboratory results” (*P*=.04), and “Generating clinician summaries” (ie, on-screen summaries; *P*=.01). Clinician responses indicated that they carried out the following tasks significantly less frequently than technical staff: “Creating new patient records” (*P*=.02) and “Updating existing patient records” (*P*=.04).

A total of 42% (37/89) of respondents stated that they used the EHR always/almost always or most of the time, as opposed to 76% (68/89) for the paper records. They *agreed or strongly agreed* >85% (71/84) of the time (Tables 1-7) with the following statements about the EMR: “I can find where to document care,” “The screen displays are easy to read,” “Content lay out is understandable,” and “It is easy to retrieve records in EHR.” For the statement “It is easy to correct errors in EHR,” agreement was 72% (64/89).

Respondents *agreed or strongly agreed* >90% (81/90) of the time that “the EHR makes it easier to manage patients’ medical file and patient’s medical follow up,” “the EHR makes it easier to make informed decisions,” “the EHR is worth the time and energy to use,” and “quality of information has improved due to the EHR.” For the statement “the EHR makes it easier to exchange patient information with other health care providers,” agreement was 71% (64/90). For the statement “the EHR provides useful alerts and reminders,” agreement was 78% (64/82) with significantly stronger agreement in the intervention sites (*P*=.01).

Answers to questions on technical and user support received mixed responses. Respondents *agreed or strongly agreed* with these questions with the following scores: “It is easy to report problems with the EHR,” 71% (63/89); “I get feedback when I report errors or problems,” 60% (53/89); “Effective help is available with the EHR,” 55% (49/89); “I use EHR because of the proportion of coworkers who use it,” 41% (35/86); “My supervisor is very supportive of EHR use on the job,” 75% (67/89); and “In general, the MOH supported the use of EHR,” 93% (84/90).

### Infrastructure

Infrastructure problems were a significant issue (Tables 1-7). The following were stated to be available *always/almost always* or *most of the occasions*: a computer when you need the EHR (77/89, 87%), grid power (74/90, 82%), wired internet connectivity (56/89, 63%), cellular internet (41/85, 48%), and a backup generator (30/88, 34%). For the question “How often can you count on EHR to be up and available*?*” response was 75% (66/88), with 18% (16/88) saying it was available about half the time and 7% (6/88) almost never.

Table 8 shows the analysis of free-text comments. The most frequent responses to the question “What are three functions you like about the electronic medical record?” were “to get client data easily and/or quickly” (62/90, 69%), “it helps to generate reliable reports in a short time” (39/90, 43%), “it stores client information safely and/or securely” (31/90, 34%), and “it helps to monitor clients on a daily basis” (20/90, 22%). In response to the question “What are three functions you do not like about the electronic medical record?” most frequent comments were “often unstable or blocked” (20/90, 22%), “hard to correct errors or unsubscribe patients” (11/90, 12%), “cannot work with OpenMRS outside the health facility/not online” (9/90, 10%), and “poor internet” (6/90, 7%).

**Table 8:** Responses to free-text questions on user likes and dislikes (n=90).

Question, themes, and example comments				Value, n (%)
**What are 3 functions you like about the electronic medical record?**				
	**Supports accessible and safe patient record keeping**			
		Helps users to get client data easily and/or quickly	62 (69)	
		Stores client information safely and/or securely	31 (34)	
	**Supports patient care by providing needed information on the patient**			
		Provides alerts	6 (6)	
	**Makes managing patient data easier**			
		“Simplifies my daily work”	14 (16)	
	**Helps to generate reports**			
		Support generation of reports reliably and in a short time	39 (43)	
	**Example comments**			
		“It provides an alert regarding viral loads, CD4.” (intervention site)	N/A^a^	
		“When it is well manipulated can reduce workload in the service.”	N/A	
		“It indicates missing information in the client’s file.”	N/A	
		“To identify client that do not respect their appointment.”	N/A	
		“Number of lost follow up.”	N/A	
**What are 3 functions you do not like about the electronic medical record?**				
	**System stability or unavailability**			
		Often unstable or blocked	20 (22)	
		Lack of technical support	7 (8)	
		Poor internet connection	6 (7)	
	**Lack of updates for key functionality or metadata**			
		Lack of drugs listed in formulary	3 (3)	
	**Lack of connectivity beyond individual health facilities**			
		Cannot work with OpenMRS outside the health facility/not on the internet	9 (10)	
		Unable to track patient transfers	4 (4)	
	**Error correction/editing**			
		Hard to correct errors	7 (8)	
		Cannot unsubscribe patients	2 (2)	
	**Example comments**			
		“There are few nurses that use OpenMRS efficiently.”	N/A	
		“You cannot use OpenMRS out of working site.”	N/A	
		“I like OpenMRS but this new version there some information that cannot provide.”	N/A	
		“I like OpenMRS but this new version there some information that cannot provide.”	N/A	
		“Blockage of OpenMRS affects my daily performance.”	N/A	

^a^N/A: not applicable.

## Discussion

### Principal Findings

Overall, the results suggest that most users of OpenMRS at Rwanda MoH health centers perceive the EHR as a valuable tool for patient care and reporting activities. The responses showed a high level of EHR use and acceptability across most health centers despite the challenges of implementing EHR systems in these environments. This finding provides foundational evidence to implementers who have an urgent need to understand how well EHRs can be scaled up to hundreds or thousands of health facilities (addressing objectives on performance and scalability). An unusual feature of the Rwanda OpenMRS implementation is the long interval since the original deployment. Some MoH health centers have used the EHR continuously for 8 or 9 years, with no major upgrades in control sites for >5 years. Therefore, this study allows assessment of the long-term performance of the EHR by typical users (objective on sustainability). Such data are not available from any existing studies that we are aware of, which have mostly focused on larger hospitals in more controlled settings with better infrastructure [11,22] or small numbers of test sites.

Responses to the 2 groups of questions most relevant to the features of the enhanced EHR package showed, in the intervention sites, more frequent use of core clinical tools, including updating records, using patient summaries, and viewing laboratory results. Significantly more respondents in the intervention sites agreed that “The electronic medical record provides useful alerts and reminders,” indicating support for more advanced EHR features added in the enhanced EHR.

There were some differences in the level of EHR use between clinicians and technical staff, including core clinical activities such as creating and updating records. Clinicians, as expected, had less technical experience and were significantly less likely to use computers outside work or access the internet for a range of applications. These findings indicate the need for further improvements in usability and workflow and in both IT and EHR training for clinicians.

It is important to note that recent versions of OpenMRS have greatly improved user interfaces and general functionality [23,24] and are expected to have significantly higher scores for usability and overall satisfaction. An up-to-date version of OpenMRS was implemented in Rwandan district hospitals in 2020/2021.

### Limitations

This survey was conducted through structured interviews with all participants. The less confidential nature of interviews compared with a web-based survey may have increased *desirability bias* as staff were aware that the study was endorsed by the MoH. There was a strong positive response on the question of MoH support and on statements that the effort to enter data and use the EHR was worthwhile. However, on other questions such as infrastructure, including power and internet connectivity, and availability of technical support, participants were more mixed in their responses, and for the question “I am generally not concerned making errors in EHR” they were clearly prepared to admit that there were problems. Many made clear that they had challenges with using the EHR, and clinicians would appear to rely on data managers and other technical staff to assist with many activities. Free-text comments provided critical insights into the actual experiences of staff, along with many other issues related to usability, use, and the need for training. The lack of significant differences in the experiences of the clinicians and technical staff regarding many questions may be partly due to the survey not being powered to show small differences between these groups. Another limitation was that the 112 sites selected for the broader study had better hardware and evidence of more consistent data entry than the others; therefore, EMR implementations described here may perform better than the full set of EMR sites in Rwanda.

### Comparison With Previous Work

Previous studies of EHR users in LMICs have identified a range of experiences. Ojo [25] used the Delone McLean Information Success Model in a study of EHR users in hospitals in Nigeria and showed that system quality and use were the most important in determining EHR success [25]. Tilahun and Fritz [26] conducted a similar study on the experience of users with an EHR in hospitals in Ethiopia. Compared with the survey in this study, they showed high levels of dissatisfaction with the EHR and low use levels owing to poor service quality (power infrastructure, user support, training, and lack of computers in the wards) and the need for double entry of data into the EHR and paper records (also a problem in Rwanda) [26]. A survey of the OpenClinic EHR users at the Kigali University Teaching Hospital in Rwanda showed strongly positive user comments on satisfaction and perception of data quality and usability compared with paper records [11].

### Conclusions

This survey provides evidence that EHR systems have become an accepted component of HIV care delivery in Rwanda. Staff were generally supportive of the system, although most wanted further training, technical support, and better power and network infrastructure. Staff at intervention sites were more likely to use or have positive experiences of key functionality that was improved in the enhanced EHR. As this survey is part of a larger evaluation study, the responses will be compared with results from key informant interviews, the costing and data quality studies, monitoring of server performance and use, and clinical impact in the cluster RCT. Further surveys are planned for other large-scale rollouts of OpenMRS in low-income settings, building on the survey form and findings in this study. The results are likely to be generalized to similar EHR systems in low-income settings if they are well tailored to the clinical needs and workflow. They are also highly relevant to the critical need for systems to support accurate, timely, and analyzable primary care data on patients in remote and very underserved clinics in low-income countries, replacing basic tools such as paper registers. This should improve the clinical documentation, care, reporting, and tracking of disease outbreaks, including COVID-19.

### Data Availability

The data underlying this paper cannot be shared publicly because of the need for privacy of the individuals who participated in the study. The data will be shared upon reasonable request with the corresponding author.

## Appendix

Multimedia Appendix 1 Survey form in English and Kinyarwanda.

## Abbreviations

EHR: electronic health record
EMR: electronic medical record
IT: information technology
LMIC: low- and middle-income country
MoH: ministry of health
RCT: randomized controlled trial
