# Supplementary material for: User Perceptions and Use of an Enhanced Electronic Health Record in Rwanda With and Without Clinical Alerts: Cross-sectional Survey
Source: JMIR Med Inform. 2022 May 3;10(5):e32305. doi: 10.2196/32305 (PMC9115652; doi:10.2196/32305)
Supplement: Multimedia Appendix 1 [file medinform_v10i5e32305_app1.docx]

**End User OpenMRS Usability Survey**

**Ibibazo byerekeye ikoreshwa ya OpenMRS kubayikoresha**

| **Location/Aho ubarizwa** | | | |
| --- | --- | --- | --- |
| **Site Name/Izina ry’ikigo** |  | | |
| **Site Number/ Numero y’ikigo** |  | | |
| **Respondent/Usubiza** | | | |
| **Respondent’s ID Number/**  **Numero iranga usubiza** |  | | |
| **Interviewer/Ubaza** | | | |
| **Date/Italiki** |  | | |
| **Interviewer’s Name/**  **Izina ry’ubaza** |  | | |
| **Language/Ururimi** | | | |
| **Language of Questionnaire/ Ururimi rw’icyongereza** |  | **Native Language of Respondent/**  **Ururimi rw’ikinyarwanda** |  |
| **Language of Interview** |  |  | |
| **Check for Completion/Kugenzura ko ibibazo byuzuye** | | | |
| **Check Performed By/ Igenzura ryakozwe na:** | Name/Izina: | | Date/Italiki: |

**Introduction**

*This survey is designed to help us understand your experience with the electronic medical record at your clinic. The information that you and other staff give us will help to improve the record system, training and infrastructure in the future. Your answers will not be shared with anyone outside the research team and we would greatly appreciate your honesty when answering the questions.*

**Intangiririro**

Iri bazwa rigamije kudufasha kumva neza uko mukoresha OpenMRS ku kigo cyanyu. Amakuru muduha azadufasha kunoza system, amahugurwa muhabwa ndetse nibikoresho bikenerwa mugihe kizaza. Ibisubizo byawe ntibizigera bisangizwa undi utari muri ubu bushakashatsi kandi turagusaba kutubwiza ukuri mugihe usubiza ibi bibazo.

**Section A. Demographics and Technology Use**

**Igice cya A. Ibiranga usubiza n’uko akoresha ikoranabuhanga**

*First, we’d like to ask you some questions about your background.*

*Icyambere, turifuza kukubaza ibibazo bikwerekeyeho.*

1. What is your job title?/ Ukora iki
   1. Nurse/ Umuforomo
   2. Doctor/Umuganga
   3. Data Manager/Ushinzwe amakuru
   4. IT Officer/ Umukozi ushinzwe ikoranabuhanga

99 Other/Ibindi *[please fill in/Uzuza hano]:* ___________________________

1. In what year did you first start working here?/Watangiye gukorera hano mu wuhe mwaka? ________________
2. How old are you?/Ufite imyaka ingahe? ______ years old/ imyaka ____ y’ubukure
3. What is your gender?/Igitsina cyawe?
4. Female/ Gore
5. Male/Gabo
6. What is the level of school you attended?/ Amashuri wize
7. No education/ nta mashuri nize
8. Some primar/ amashuri abanza
9. Completed primary/ narangije amashuri abanza
10. Some secondary/ Nize amashuri yisumbuye
11. Completed secondary/ Narangije amashuri yisumbuye
12. More than secondary/ mfite amashuri ari hejuru y’ayisumbuye
13. How often do you do the following activities?/Ni kangahe ukora ibi bikurikira?

|  |  | Less than once a month/munsi ya rimwe mu kwezi | Monthly/buri kweizi | Weekly/ buri cyumweru | Daily/ buri munisi | More than once a day/ insure irenze imwe ku munsi |
| --- | --- | --- | --- | --- | --- | --- |
| a) | Use a mobile phone to send text messages  Gukoresha telephone mobile kohereza ubutumwa | ☐  1 | ☐  2 | ☐  3 | ☐  4 | ☐  5 |
| b) | Use a mobile phone to access email, internet, WhatsApp, or Facebook/  Gukoresha telephone mobile kuri murandasi, whattsap cyangwa facebook | ☐  1 | ☐  2 | ☐  3 | ☐  4 | ☐  5 |
| c) | Use a computer outside of work/ Gukoresha imashini Atari mukazi | ☐  1 | ☐  2 | ☐  3 | ☐  4 | ☐  5 |
| d) | Access the internet to check email, go to websites, or any other internet activities/ Gukoresha telephone kubindi nka murandasi | ☐  1 | ☐  2 | ☐  3 | ☐  4 | ☐  5 |

*Now we’d like to ask you some questions about your use and experience with the electronic medical record system.*

*Noneho tugiye kukubaza ibibazo byerekeye uko ukoresha ndetse n’ubunararibonye mu gukoresha OpenMRS*

**Section B. Training on OpenMRS/ Igice cya B. Amahugurwa**

1. How many months has it been since you first used the electronic medical record?/ haciye amezi angahe kuva utangiye gukoresha OpenMRS?

__________ month(s)/ Amezi

1. How many separate trainings have you received on the electronic medical record system? For example, one 3-day long training would count only as a single training./ Waba warakoze amahugurwa ya OpenMRS inshuro zingahe? Urugero amahugura wakoze iminsi itatu bivuze inshuro imwe.

____________ training(s)/amahugurwa

1. In total, how many days of training did you receive on the electronic medical record?/ Ukoze igiteranyo, waba warahuguwe iminsi ingahe mu mahugurwa wakoze kuri OpenMRS.

__________ day(s)/Iminsi

1. Please indicate the degree to which you agree with the following statements/Garagaza ikigero wemera ibi bikurikira:

|  |  | Strongly disagree/Simbyemera na gato | Disagree/simbyemera | Neutral/ndifashe | Agree/Ndabyemera | Strongly agree/Ndabyemera cyane | | Not applicable/Nibijyanye |
| --- | --- | --- | --- | --- | --- | --- | --- | --- |
| a) | The training I received relating to the electronic medical record was effective.  Amahugurwa nabonye yerekeye OpenMRS yari ingirakamaro | ☐  1 | ☐  2 | ☐  3 | ☐  4 | ☐  5 | ☐  98 | |
| b) | In general, I am not concerned about making errors in the electronic medical record./Muri rusange nacyo bimbwiye gukora amakosa muri OpenMRS | ☐  1 | ☐  2 | ☐  3 | ☐  4 | ☐  5 | ☐  98 | |
| c) | I am confident using the electronic medical record./ Ndemera ntashidikanya ko nshobora gukoresha OpenMRS | ☐  1 | ☐  2 | ☐  3 | ☐  4 | ☐  5 | ☐  98 | |

1. Do you have any other comments on the training you received on the electronic medical record? *[Please record here]/* Waba ufite ikindi wifuza kuvuga kubyerekeye amahugurwa wabonye kuri OpenMRS? *[Byandike hano]*

**Section C. Frequency of Use of OpenMRS/ Inshuro ukoresha OpenMRS**

1. Please indicate how often you use the electronic medical record to assist you with the following tasks/ Garagaza insure ukoresha OpenMRS mu kugufasha gukora ibi bikurikira.

|  |  | Never/ almost never/Ntanarimwe | Seldom/gake gashoboka | About half of the occasions/Rimwe na rimwe | Most of the occasions/Akenshi | Always/ almost always/Buri gihe cyane | Our EMR doesn’t support this task/OpenMRS ntikora ibyo | This task doesn’t apply to me/Ibyo ntibindeba |
| --- | --- | --- | --- | --- | --- | --- | --- | --- |
| a) | Creating new patient records/ Kwandika umurwayi mushya | ☐  1 | ☐  2 | ☐  3 | ☐  4 | ☐  5 | ☐  98 | ☐  99 |
| b) | Updating existing patient records/ Kusuzuza amakuru mashya ku murwayi usanzwe muri OpenMRS | ☐  1 | ☐  2 | ☐  3 | ☐  4 | ☐  5 | ☐  98 | ☐  99 |
| c) | Generating patient summaries before visits/Gukora incamake y’ibyerekeye umurwayi | ☐  1 | ☐  2 | ☐  3 | ☐  4 | ☐  5 | ☐  98 | ☐  99 |
| d) | Reviewing previous patient encounters/Kureba ibyakorewe umurwayi | ☐  1 | ☐  2 | ☐  3 | ☐  4 | ☐  5 | ☐  98 | ☐  99 |
| e) | Ordering laboratory analyses/ Gusaba ibizamini bya Laboratwari | ☐  1 | ☐  2 | ☐  3 | ☐  4 | ☐  5 | ☐  98 | ☐  99 |
| f) | Viewing laboratory results/Kureba ibizamini bya laboratwari | ☐  1 | ☐  2 | ☐  3 | ☐  4 | ☐  5 | ☐  98 | ☐  99 |
| g) | Following test results over time/Gukurikirana ibisubizo by’ibizamini | ☐  1 | ☐  2 | ☐  3 | ☐  4 | ☐  5 | ☐  98 | ☐  99 |
| h) | Ordering medicine/Gusaba imiti | ☐  1 | ☐  2 | ☐  3 | ☐  4 | ☐  5 | ☐  98 | ☐  99 |
| i) | Generating pharmacy reports/Gukora raporo ya farumasi | ☐  1 | ☐  2 | ☐  3 | ☐  4 | ☐  5 | ☐  98 | ☐  99 |
| j) | Generating automatic reports/gukora raporo zisanzwemo | ☐  1 | ☐  2 | ☐  3 | ☐  4 | ☐  5 | ☐  98 | ☐  99 |
| k) | Generating adhoc reports (e.g., quarterly or TracNET reports)/Gukora raporo ziba zikenewe (urugero tracnet) | ☐  1 | ☐  2 | ☐  3 | ☐  4 | ☐  5 | ☐  98 | ☐  99 |
| l) | Generating consult sheets/ Gusaba raporo igufasha kuvura | ☐  1 | ☐  2 | ☐  3 | ☐  4 | ☐  5 | ☐  98 | ☐  99 |
| m) | Generating clinician summaries/Gusaba incamake ya muganga | ☐  1 | ☐  2 | ☐  3 | ☐  4 | ☐  5 | ☐  98 | ☐  99 |
| n) | Referring patients to another health centre/ kohereza umurwayi ku kindi kigo | ☐  1 | ☐  2 | ☐  3 | ☐  4 | ☐  5 | ☐  98 | ☐  99 |

|  |  | Never/ almost never/Ntanarimwe | Seldom/gake gashoboka | About half of the occasions/Rimwe na rimwe | Most of the occasions/Akenshi | Always/ almost always/Buri gihe cyane | Not applicable/ntibijyanye |
| --- | --- | --- | --- | --- | --- | --- | --- |
| 13) | All considered, how often do you use the electronic medical record as an information source in your clinical work?/Dushyize muri rusange ni kangahe ukoresha OpenMRS nkuburyo bwo kubona amakuru mu kazi kawe ku kigo | ☐  1 | ☐  2 | ☐  3 | ☐  4 | ☐  5 | ☐  98 |
| 14) | All considered, how often do you use paper-based medical records as an information source in your clinical work?/ Dushyize muri rusange ni kangahe ukoresha amafishi y’abarwayi nkuburyo bwo kubona amakuru mu kazi kawe ku kigo | ☐  1 | ☐  2 | ☐  3 | ☐  4 | ☐  5 | ☐  98 |

15) Do you have any other comments about how often you use the electronic medical record? *[Please record here]* Waba ufite ikindi wifuza kuvuga kubyerekeye inshuro ukoresha OpenMRS? *[Byandike hano]*

**Section D. Ease of Use of OpenMRS/ Uburyo byoroshye gukoresha OpenMRS**

16) Please tell us the degree to which you agree or disagree with the following statements about the electronic medical record*./ Tugaragarize ikigero wemera cyangwa uhakana ibi bikurikira byerekeye OpenMRS*

|  |  | Strongly disagree/Simbyemera na gato | Disagree/simbyemera | Neutral/ndifashe | Agree/Ndabyemera | Strongly agree/Ndabyemera cyane | | Not applicable/Nibijyanye | |
| --- | --- | --- | --- | --- | --- | --- | --- | --- | --- |
| a) | I am able to find where I need to document patient care./Nshoboye kubona aho nandika uko navuye umurwayi | ☐  1 | ☐  2 | ☐  3 | ☐  4 | ☐  5 | ☐  98 | |  |
| b) | In general, it is easy to correct errors in the electronic medical record./Muri rusange biroroshye gukosora amakosa muri OpenMRS | ☐  1 | ☐  2 | ☐  3 | ☐  4 | ☐  5 | ☐  98 | |  |
| c) | In general, the screen display is easy to read./ Muri rusange uko ekara igaragara biroroshye kuyisoma | ☐  1 | ☐  2 | ☐  3 | ☐  4 | ☐  5 | ☐  98 | |  |
| d) | The content is laid out in an understandable way./ Ibikubiyemo bikoze kuburyo byumvikana | ☐  1 | ☐  2 | ☐  3 | ☐  4 | ☐  5 | ☐  98 | |  |
| e) | It is easy to retrieve patient records in the electronic medical record system./Biroroshye kubona amakuru y’umurwayi muri OpenMRS | ☐  1 | ☐  2 | ☐  3 | ☐  4 | ☐  5 | ☐  98 | |  |

1. Do you have any other comments or feedback about how easy or hard it is to use the electronic medical record? *[Please record here]* Waba ufite ikindi wifuza kuvuga kubyerekeye uburyo byoroshye cyangwa bikomeye gukoresha OpenMRS? *[Byandike hano]*

**Section E. Usefulness of OpenMRS/ Igice cya E. Umumaro wa OpenMRS**

18) Please tell us the degree to which you agree or disagree with the following statements about the electronic medical record*. / Tubwire ikigero wemeraho cyangwa uhakana ibi bikurikira byerekeye OpenMRS.*

|  | | Strongly disagree/Simbyemera na gato | Disagree/simbyemera | Neutral/ndifashe | Agree/Ndabyemera | Strongly agree/Ndabyemera cyane | Not applicable/Nibijyanye |
| --- | --- | --- | --- | --- | --- | --- | --- |
| a) | The electronic medical record provides useful alerts and reminders./OpenMRS itanga aleriti zifite umumaro | ☐  1 | ☐  2 | ☐  3 | ☐  4 | ☐  5 | ☐  98 |
| b) | The electronic medical record makes it easier to manage patients./OpenMRS yoroshya gukurikirana abarwayi | ☐  1 | ☐  2 | ☐  3 | ☐  4 | ☐  5 | ☐  98 |
| c) | The electronic medical record makes it easier to make informed decisions./OpenMRS yoroshya gufata ibyemezo bishingiye ku makuru | ☐  1 | ☐  2 | ☐  3 | ☐  4 | ☐  5 | ☐  98 |
| d) | The electronic medical record makes it easier to exchange patient information with other health care providers./OpenMRS yoroshya gusangira amakuru nabandi baganga/foromo | ☐  1 | ☐  2 | ☐  3 | ☐  4 | ☐  5 | ☐  98 |
| e) | The electronic medical record is worth the time and energy to use it./Igihe n’imbaraga bisaba gukoresha OpenMRS bifite agaciro | ☐  1 | ☐  2 | ☐  3 | ☐  4 | ☐  5 | ☐  98 |
| f) | The quality of information has improved due to the electronic medical record./Ireme ry’amakuru ryariyongereye kuberaOpenMRS | ☐  1 | ☐  2 | ☐  3 | ☐  4 | ☐  5 | ☐  98 |

1. Do you have any other comments or feedback about the usefulness of the electronic medical record? *[Please record here] Waba ufite ikindi wakongeraho kubyerekeye umumaro wa OpenMRS?*

**Section F. User Support for OpenMRS/ Igice F. Ubufasha buhabwa abakoresha OpenMRS**

20) Please tell us the degree to which you agree or disagree with the following statements about the electronic medical record*./Tubwire ikigero wemeraho cyangwa uhakana ibi bikurikira byerekeye OpenMRS.*

|  |  | Strongly disagree/Simbyemera na gato | Disagree/simbyemera | Neutral/ndifashe | Agree/Ndabyemera | Strongly agree/Ndabyemera cyane | | Not applicable/Nibijyanye |  |
| --- | --- | --- | --- | --- | --- | --- | --- | --- | --- |
| a) | It is easy to report problems with the electronic medical record./Biroroshye kumenyesha ibibazo bya OpenMRS | ☐  1 | ☐  2 | ☐  3 | ☐  4 | ☐  5 | ☐  98 | | |
| b) | I get feedback when I report errors or problems with the electronic medical record./ Mbona igisubizo iyo menyesheje ikosa cyangwa ibibazo bya OpenMRS | ☐  1 | ☐  2 | ☐  3 | ☐  4 | ☐  5 | ☐  98 | | |
| c) | Effective help is available when I experience problems with the electronic medical record./ Ubufasha bukwiye buraboneka iyo ngize ikibazo muri OpenMRS | ☐  1 | ☐  2 | ☐  3 | ☐  4 | ☐  5 | ☐  98 | | |
| d) | I use the electronic medical record because of the proportion of my coworkers who use it./Nkoresha OpenMRS kubera uruhare rwa bagenzi banjye dukorana bayikoresha | ☐  1 | ☐  2 | ☐  3 | ☐  4 | ☐  5 | ☐  98 | | |
| e) | My supervisor is very supportive of the use of the electronic medical record for my job./ Umuyobozi wanjye aramfasha cyane kubyerekeye gukoresha OpenMRS mukazi kanjye | ☐  1 | ☐  2 | ☐  3 | ☐  4 | ☐  5 | ☐  98 | | |
| f) | In general, the Ministry of Health has supported the use of the electronic medical record./ Muri rusange minisiteri y’ubuzima yafashije ikoreshwa rya OpenMRS | ☐  1 | ☐  2 | ☐  3 | ☐  4 | ☐  5 | ☐  98 | | |

1. Do you have any other comments or feedback about support for the electronic medical record? *[Please record here]/ Waba ufite ikindi wavuga kubyerekeye kubufasha ubona bwa OpenMRS*

**Section G. Infrastructure for OpenMRS/ Ibikorwa remezo bya OpenMRS**

22) Please indicate how often you experience the following:/ Garagaza inshuro uhura nibi bikurikira

|  |  | Never/ almost never/Ntanarimwe | Seldom/gake gashoboka | About half of the occasions/Rimwe na rimwe | Most of the occasions/Akenshi | Always/ almost always/Buri gihe cyane | Not applicable/ntibijyanye |
| --- | --- | --- | --- | --- | --- | --- | --- |
| a) | How often can you count on the electronic medical record to be up and available?/Ni ryari ushobora kwizere ko OpenMRS yaba ikora kandi wayibona | ☐  1 | ☐  2 | ☐  3 | ☐  4 | ☐  5 | ☐  98 |
| b) | How often is grid electricity present?/Ni kangahe umuriro uba uhari | ☐  1 | ☐  2 | ☐  3 | ☐  4 | ☐  5 | ☐  98 |
| c) | How often is the backup generator available?/Ni kangahe moteri iba ihari mugihe nta muriro | ☐  1 | ☐  2 | ☐  3 | ☐  4 | ☐  5 | ☐  98 |
| d) | How often is there internet?/ Ni kangahe uba ufite internet | ☐  1 | ☐  2 | ☐  3 | ☐  4 | ☐  5 | ☐  98 |
| e) | How often is there cellular network coverage?/ Nikangahe uba ufite modem | ☐  1 | ☐  2 | ☐  3 | ☐  4 | ☐  5 | ☐  98 |
| f) | How often is a computer available when you need to use the electronic medical record?/ Nikangahe machine iba ihari igihe ushaka gukoresha OpenMRS | ☐  1 | ☐  2 | ☐  3 | ☐  4 | ☐  5 | ☐  98 |
| g) | How often is the electronic medical record very slow?/Ni kangahe OpenMRS iba igenda gahoro | ☐  1 | ☐  2 | ☐  3 | ☐  4 | ☐  5 | ☐  98 |

23) Do you have any other comments or feedback about the infrastructure for the electronic medical record? *[Please record here]/ Waba ufite ikindi wifuza kutubwira ku byerekeye ibikorwa remezo bya OpenMRS?*

**Section H. Functions of OpenMRS/ Igice cya F. Umumaro wa OpenMRS**

24) What are three functions you like about the electronic medical record? /Tubwire ibintu bitatu ukunda kuri OpenMRS

1. _________________________________

2. _________________________________

3. _________________________________

25) What are three functions you do not like about the electronic medical record? //Tubwire ibintu bitatu udakunda kuri OpenMRS

1. _________________________________

2. _________________________________

3. _________________________________

26) Do you have any other comments about the electronic medical record or HIV monitoring you would like to share? *[Please record here]/ Waba ufite ikindi gitekerezo wifuza kudusangiza kubyerekeye OpenMRS cyangwa uko wakurikirana abarwayi muri porogaramu ya SIDA?*

*Thank you for taking the time to complete this survey! Your answers will help to improve the electronic medical record system in the future./ Tugushimiye igihe wafashe cyo kuzuza ibi bibazo! Ibisubizo bizafasha kunoza OpenMRS mugihe kiri imbere.*
